# Complement Evasion Mediated by Enhancement of Captured Factor H: Implications for Protection of Self-Surfaces from Complement

**DOI:** 10.4049/jimmunol.1501388

**Published:** 2015-10-12

**Authors:** Andrew P. Herbert, Elisavet Makou, Zhuo A. Chen, Heather Kerr, Anna Richards, Juri Rappsilber, Paul N. Barlow

**Affiliations:** * School of Chemistry, University of Edinburgh, Edinburgh EH9 3FJ, United Kingdom;; † School of Biological Sciences, University of Edinburgh, Edinburgh EH9 3BF, United Kingdom; and; ‡ Queen’s Medical Research Institute, Edinburgh EH16 4TJ, United Kingdom

## Abstract

In an attempt to evade annihilation by the vertebrate complement system, many microbes capture factor H (FH), the key soluble complement-regulating protein in human plasma. However, FH is normally an active complement suppressor exclusively on self-surfaces and this selective action of FH is pivotal to self versus non-self discrimination by the complement system. We investigated whether the bacterially captured FH becomes functionally enhanced and, if so, how this is achieved at a structural level. We found, using site-directed and truncation mutagenesis, surface plasmon resonance, nuclear magnetic resonance spectroscopy, and cross-linking and mass spectrometry, that the N-terminal domain of *Streptococcus pneumoniae* protein PspC (PspCN) not only binds FH extraordinarily tightly but also holds it in a previously uncharacterized conformation. Functional enhancement arises from exposure of a C-terminal cryptic second binding site in FH for C3b, the activation-specific fragment of the pivotal complement component, C3. This conformational change of FH doubles its affinity for C3b and increases 5-fold its ability to accelerate decay of the binary enzyme (C3bBb) responsible for converting C3 to C3b in an amplification loop. Despite not sharing critical FH-binding residues, PspCNs from D39 and Tigr4 *S. pneumoniae* exhibit similar FH-anchoring and enhancing properties. We propose that these bacterial proteins mimic molecular markers of self-surfaces, providing a compelling hypothesis for how FH prevents complement-mediated injury to host tissue while lacking efficacy on virtually all other surfaces. In hemolysis assays with 2-aminoethylisothiouronium bromide–treated erythrocytes that recapitulate paroxysmal nocturnal hemoglobinuria, PspCN enhanced protection of cells by FH, suggesting a new paradigm for therapeutic complement suppression.

## Introduction

The vertebrate complement system is a network of plasma and cell-surface proteins. Upon activation, these proteins cooperate to selectively tag and eliminate invading organisms and immune complexes along with products of apoptosis and oxidative damage (1). Bacterial complement-evasion strategies are targets for novel antibiotics or vaccines, although therapeutic suppression of complement activation is desirable in many diverse clinical contexts (2).

**FIGURE 1.**
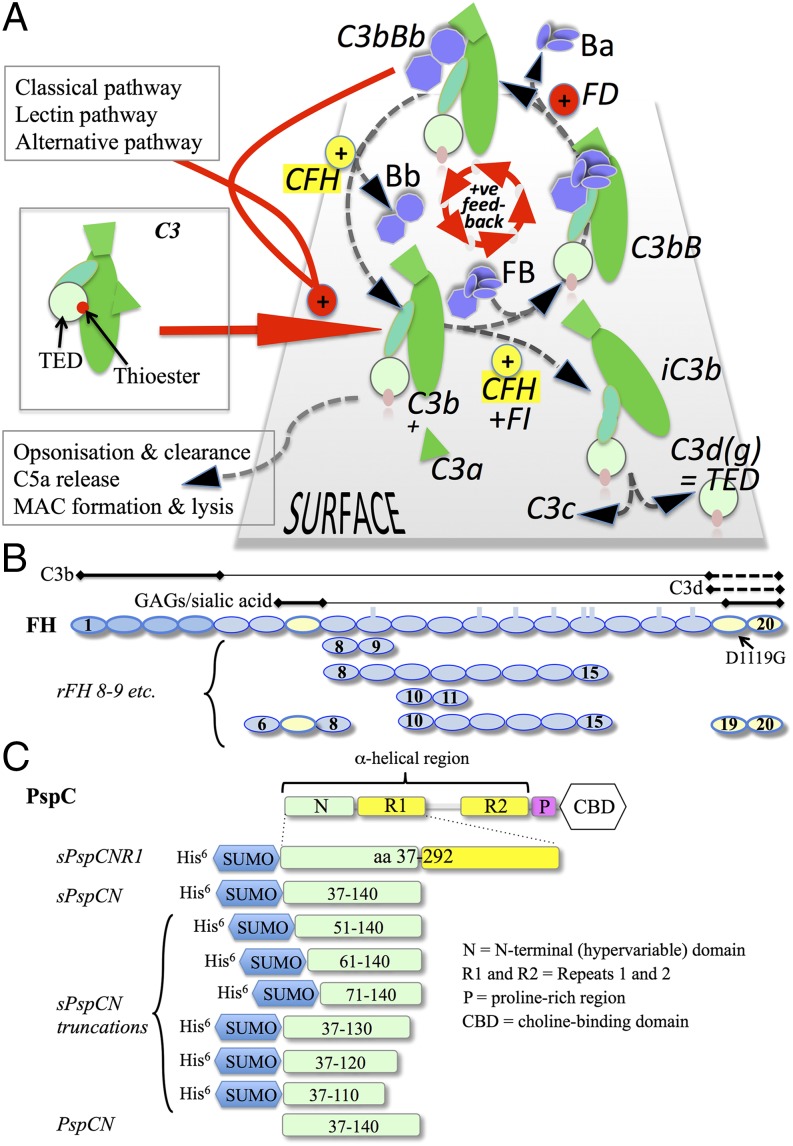
Proteins used in this study. (**A**) The alternative pathway produces, spontaneously, low levels of C3b from inert C3. Nascent C3b can attach to nearby surfaces via its TED. C3b also binds FB, forming C3bB. Factor D (FD) cleaves the FB portion of C3bB, yielding C3bBb. C3bBb converts C3 to C3b (+ C3a), establishing a positive-feedback loop for C3b production. On a self-surface, FH competes with FB for C3b binding and accelerates decay of C3bBb. C3b can be degraded to iC3b by FI with FH as cofactor and then via C3dg (+ C3c) to C3d, equating to the TED of C3b. (**B**) Ovals represent CCPs 1–20 in FH; locations of N-glycans are indicated by rectangles. Horizontal lines above the ovals designate CCPs involved in binding C3b (ovals with thicker outline), self-surface markers (pale shading), and C3d (pale shading plus thicker outline); dashed lines indicate constitutively occluded binding sites. (**C**) N-terminally His^6^-tagged SUMO fusions (s) with sequences from the N-terminal domain of D39 PspC (residue numbering includes signaling sequence) were created. The sPspCN_37–140_ or the product (PspCN) following cleavage of and removal of the fusion partner was used for most experiments.

Central to the “alternative” pathway of complement activation (3) is a positive-feedback loop that amplifies rapidly the protein C3b (Fig. 1A). In this pathway, a trickle of C3b arises spontaneously from low-level proteolysis of the abundant plasma precursor, C3. Amplification may ensue via participation of C3b in the binary complex, C3bBb. This cleaves (converts) C3 to yield additional C3b. Nascent C3b has a transient capability to bond covalently, via a thioester-containing domain (TED), to virtually any nearby surface. Thus, unless complement regulators intercede, C3b amplification kicks off and all host and foreign surfaces in the vicinity risk being coated in C3b. C3b is an opsonin and an initiator of further steps in complement, promoting inflammation and cytolysis. C3b also arises via other complement-activation pathways. Crucially, it is the selective actions of complement regulators that limit C3b amplification on a surface and thus determine the fates of all plasma-exposed cells and particles.

Factor H (FH) (4–7) is the key soluble regulator of C3b amplification. It is unique in structure, consisting entirely of 20 complement-control protein modules (CCPs) (8, 9) (Fig. 1B). Factor H inhibits C3bBb formation by competing with factor B (FB) (precursor of Bb) for binding to C3b, accelerates irreversible C3bBb decay by displacing Bb, and is a cofactor for factor I (FI)–catalyzed C3b cleavage, yielding iC3b that cannot bind FB. Subsequent iC3b cleavage ultimately yields C3d, equating to the TED of C3b (Fig. 1A). C3b degradation products, including C3d, remain covalently tethered to the surface and augment adaptive immunity. A critical property of FH is that it regulates C3b preferentially on self-surface. This underpins self versus non-self selectivity by the complement system. However, despite decades of effort, the molecular mechanism for the selective regulatory action of FH is not fully understood.

The importance of FH is highlighted by links between variations in its gene, *CFH*, and diseases (10). Responsible for ∼50% of the risk of age-related macular degeneration (11–14), one *CFH* haplotype encodes a substitution in FH CCP 7 contributing to recognition by FH of self-surface-markers, including glycosaminoglycans (GAGs) (15–18) (Fig. 1B). Mutations linked to atypical hemolytic uremic syndrome (aHUS) cluster in C-terminal CCPs 19 and 20 (19, 20). These modules participate, along with CCP 7, in self-surface binding via GAGs and sialic acids; CCPs 19 and 20 also interact with the TED of C3b and C3b degradation products, iC3b and C3d (16). CCPs 1–4 of FH bind C3b (but not iC3b/C3d), are crucial for FI cofactor and C3bBb decay-accelerating activities (21), and harbor mutations or single nucleotide polymorphisms linked to age-related macular degeneration, aHUS, and C3 glomerulonephritis (22, 23). Promising therapeutic complement-suppressing proteins incorporate CCPs 1–5 (24) or CCPs 1–4 plus CCPs 19 and 20 (25, 26). A construct consisting of CCPs 1–4 flexibly linked to CCPs 19 and 20 inhibited complement-mediated lysis of erythrocytes from paroxysmal nocturnal hemoglobinuria (PNH) patients lacking key membrane-associated regulators (27) (see below).

As chief soluble complement regulator, FH is a prime target for microbial hijack (28–30). For instance, choline-binding protein PspC (SpsA, CbpA, Hic) (Fig. 1C) from the nasopharyngeal commensal bacterium *Streptococcus pneumoniae* recruits host-derived FH from plasma and onto the bacterial surface (31–33). Invasion of blood by *S. pneumoniae* can cause pneumonia, meningitis, and septicemia. Serotype invasiveness seems to rely on bacterial ability to bind FH, although the functional activities of bacteria-bound FH were not experimentally compared with those of circulating FH. Differences in levels, or allelic variation, of PspC are likely responsible for variations in bacterial FH binding (34).

An evolutionary host-pathogen arms race may have helped to define the enigmatic relationship between the unique 20-CCP structure of FH and its key property of self-surface selectivity. Complete reliance on preferential binding by FH, to distinguish self-surfaces from foreign ones, would be wide open to exploitation via simple capture on a bacterial surface. We now show that an N-terminal domain of *S. pneumoniae* protein PspC (PspCN) not only binds FH extraordinarily tightly, but is also able to stabilize, via an allosteric effect, a previously unreported, functionally enhanced, conformation of FH in which an occluded C-terminal TED/C3d-binding site is exposed. The ability of FH to bind C3b is thereby enhanced 2-fold and its C3bBb decay-accelerating activity is improved 5-fold. This could be an example of a bacterial protein mimicking molecular markers of self-surfaces.

## Materials and Methods

### Preparation of proteins

Plasma-derived human complement proteins from Complement Technologies (Tyler, TX) were used without further purification. Recombinant human FH, with a wild-type sequence, was produced in recombinant *Pichia pastoris* in a fermentor and purified after enzymatic deglycosylation (35). Expression-optimized DNA encoding the FH(D1119G) mutant was synthesized (GeneArt–Life Technologies) and subcloned into *P. pastoris* expression vector pPICZαB. Production of pure recombinant FH (rFH; D1119G) was performed as described for rFH. A set of rFH module fragments (Fig. 1B) was prepared from recombinant *P. pastoris* as described (16).

Synthetic genes encoding the appropriate residues of PspC from D39 *S. pneumoniae* (see Fig. 1C) or 37–179 from Tigr4 *S. pneumoniae* were optimized for *Escherichia coli* (GeneArt–Life Technologies) and cloned into *E. coli* vector pE-SumoProKan (LifeSensors, Malvern, PA) then expressed in BL21 (DE3) *E. coli*. Protein production was induced by addition of isopropyl β-d-1-thiogalactopyranoside. The resultant hexa-His-SUMO–tagged proteins (see Fig. 1C) were captured on a HisTrap column (GE Healthcare) and eluted with a gradient to 0.5 M imidazole. They were further purified by size-exclusion chromatography (HiPrep Superdex 75 column, GE Healthcare) in PBS. For most experiments, the catalytic domain from SUMO-specific protease, ULP1, was used to cleave the hexa*-*His-SUMO tag from fusion constructs and the tag removed on a HisTrap column. The cleaved material was purified by size-exclusion chromatography. Homogeneity was confirmed by SDS-PAGE and mass spectrometry (MS) (data not shown).

### Measurement of FH binding to PspCN by surface plasmon resonance

Surface plasmon resonance (SPR) experiments were performed at 25°C on a Biacore T200 instrument (GE Healthcare) using 10 mM HEPES, 150 mM NaCl, 0.05% (v/v) surfactant P20 (pH 7.4) supplemented with 50 μM EDTA. Routinely, Ni^2+^ (typically ∼50 response units [RU]) and subsequently the PspC-derived proteins were immobilized on a Biacore NTA chip via N-terminal His^6^-SUMO tags (typically ∼150 RU). Solutions of plasma-derived FH, rFH, or rFH fragments were flowed over the chip (180 s, 100 μl/min) followed by a dissociation phase of 1500 s. Because effectively irreversible binding of FH occurred, the chip was regenerated between measurements by two 30-s injections of 350 mM EDTA in 1 M NaCl, then 50 mM NaOH, followed by 0.5% (w/v) SDS. Data were analyzed using the Biacore evaluation software and a 1:1 binding model. No refractive index correction or mass transport correction was applied; the drifting baseline was corrected by subtracting the signal obtained from an injection of 0 concentration of analyte.

We also observed effectively irreversible binding of FH to PspCN (after cleavage of the His^6^-SUMO tag) amine coupled to a Biacore CM5 chip, and to nontagged PspCN, with a C-terminal cysteine, thiol coupled to a Biacore C1 chip (data not shown). Use of the Ni-NTA chip, however, minimized the risks, associated with amine coupling, of heterogeneous protein deposition and masking of ligand-recognition sites. It also facilitated regeneration of the chip surface. Its disadvantages were slow ongoing leaching of His^6^-tagged SUMO fusion of D39 PspCN (sPspCN) from the surface, and the the potential for FH and some FH fragments to bind directly to divalent cations, including Ni^2+^ (36). Indeed, we observed weak binding of FH (see Supplemental Fig. 1) and moderate binding of FH 6–8 to the Ni-NTA chip surface. These difficulties were circumvented by background subtraction.

### Measurement of FH and FH:PspCN binding to C3b and C3d by SPR

In these experiments C3b (typically ∼250 RU) or C3d (typically ∼70 RU) was amine coupled to adjacent flow cells of a Biacore C1 chip (another flow cell was used for background subtraction). Solutions of FH (preincubated with or without a 2-fold molar excess of PspCN) were injected (90 s, 30 μl/min) over the chip, followed by a 400-s dissociation phase. The sensor chip was regenerated by two injections of 1 M NaCl. Data were analyzed using the Biacore evaluation software and a 1:1 binding model.

### C3bBb decay-accelerating assay

Decay-accelerating activity was measured in an SPR-based assay as described (37). In brief, ∼500 RU C3b was amine coupled to a Biacore CM5 chip, then C3 convertase (C3bBb) was assembled on the chip by a 120-s 10 μl/min injection of 500 nM FB plus 50 nM complement factor D. Formation of C3 convertase (to ∼150 RU) was monitored, then an initial dissociation phase (200 s) allowed observation of intrinsic convertase decay (Bb loss). Subsequently, FH or FH:PspCN was injected (10 μl/min for 120 s), followed by a further dissociation phase. The chip was regenerated between cycles by a 30-s injection of 0.2 μM FH and two 30-s injections of 1 M NaCl.

### FI cofactor assay

The activity of FH as a cofactor for FI-catalyzed C3b cleavage was measured by detection of its initial α′-chain proteolytic products (63 and 39 kDa) following SDS-PAGE. The non-cleaved β-chain served as an internal control. The initial 16-μl reaction mixture contained 1.7 μM C3b, 14 nM FI, and a range of concentrations (80, 40, 20, 10 nM) of FH, either alone or with a 10-fold molar excess of PspCN. The reaction mixture was incubated for 30 min (37°C), then stopped by addition of SDS-PAGE loading buffer. The mixture was boiled for 300 s and subjected to PAGE.

### Hemolytic assay

Sheep and rabbit whole blood was from TCS Biosciences (Buckingham, U.K.). FH-depleted normal human serum (FHΔNHS) was from Complement Technology. Hemolysis assays were performed in 96-well plates as described (38), except HEPES replaced veronal buffer. Erythrocytes were washed thrice with 20 mM HEPES, 145 mM NaCl, 10 mM EDTA, and 0.1% (w/v) gelatin (porcine skin, Fluka) (pH 7.3) (buffer H1), and a further three times with 20 mM HEPES, 145 mM NaCl, 100 μM EDTA, and 0.1% (w/v) gelatin (pH 7.3) (buffer H2). Erythrocytes were used at a concentration that gave a final absorbance at 412 nm of 1.0 (rabbit) or 1.2 (sheep) when lysed completely in water. FHΔNHS was reconstituted with FH to 2 μM and then combined, on ice, and made up to the final concentrations indicated, with erythrocytes and buffer H2 yielding a 45 μl final volume. Lysis was initiated by addition of 5 μl 50 mM Mg^2+^, 50 mM ethylene glycol tetra-acetic acid (pH 7.3). The reaction was incubated (37°C, 30 min) and then quenched with 200 μl buffer H1. Nonlysed cells and cell debris were pelleted (1500 × *g*, 10 min, 4°C), then 100 μl supernatant was taken for A_412_ measurement.

### Protection against hemolysis of “PNH-like” erythrocyte

Human erythrocytes lacking GPI-linked proteins (PNH-like) were prepared according to a modified version of the method of Ezzell et al. (39). Whole human blood was obtained by phlebotomy and stored in Alsever’s solution (4°C). Human blood samples were obtained for this study exclusively from healthy adult volunteers who provided written informed consent, in accordance with the Research Ethics Framework of the College of Science and Engineering of the University of Edinburgh and guidance from the University of Edinburgh’s Research Ethics Committee. Cells were washed three times with wash buffer (20 mM HEPES, 145 mM NaCl, 10 mM EDTA, 0.1% [w/v] gelatin [pH 7.3]) and then resuspended in PBS. After each wash step the top 10% of the cell pellet was aspirated to remove the white cell–containing buffy coat. For removal of decay-accelerating factor and other GPI-linked proteins, a 1-ml aliquot of packed cells was added to an 8% (w/v) solution of 2-aminoethylisothiouronium bromide (AET, Sigma-Aldrich) solution that had been titrated to pH 8.0 with HCl. The cell suspension was incubated for 9 min (37°C) with constant agitation, after which the resultant PNH-like cells were washed three times with wash buffer containing no EDTA.

For hemolysis assays, the PNH-like cells were washed with 20 mM HEPES, 145 mM NaCl, 0.1% (w/v) gelatin (pH 6.4) and then incubated (37°C) with acidified NHS, 5 mM magnesium EGTA, and the appropriate concentration of either plasma-purified FH or purified rPspCN, in a total reaction volume of 50 μl. After 30 min the reaction was quenched by the addition of 200 μl ice-cold buffer (20 mM HEPES, 145 mM NaCl, 10 mM EDTA, 0.1% [w/v] gelatin [pH 7.3]). The cells and cell debris were removed by centrifugation (3000 × *g*, 10 min, 4°C), after which a 100-μl aliquot was removed and its absorbance at 412 nm recorded.

### Assessment of oligomeric status

For determination of oligomeric status, samples (4.6 μM in PBS) of FH, PspCN, or of a 1:1 complex of these proteins were characterized by size-exclusion chromatography–multiple-angle light scattering. Following passage through a Superose12 10/300 GL column (GE Healthcare) operating at 0.4 ml min^−1^, the eluate was subjected to measurements of absorbance, refractive index, and light scattering using a Dawn Heleos II detector (Wyatt Technology). Data were analyzed by ASTRA software. Samples as above were also subjected to analysis using a Beckman XL-A ultracentrifuge with an An-50 Ti eight-hole rotor (Beckman Instruments, Fullerton, CA). Velocity sedimentation analysis was performed at 40,000 rpm (25°C). The sedimenting boundary was monitored every 90 s until completion. Data were processed using sedimentation coefficient distributions analysis in SEDFIT (40).

### Nuclear magnetic resonance spectroscopy

Spectra were acquired on an Avance II 800 MHz spectrometer (Bruker) using a 5-mm TCI CryoProbe (Bruker). Data were processed using TopSpin 3.1 (Bruker) and analyzed using CcpNmr analysis software (41). Samples of ^15^N-FH 8–9 and ^15^N-PspCN were used to optimize conditions by varying NaCl from 0 to 150 mM (pH from 4 to 6.5) and temperature from 15 to 70°C. Subsequently, 40 μM ^15^N-FH 8–9 in 20 mM potassium phosphate (pH 6.2) was used to record a ^1^H,^15^N HSQC spectrum at 310 K, then PspCN was added to 100 μM and the spectrum was rerecorded. Separately, 30 μM ^15^N-PspCN in 20 mM potassium phosphate (pH 6.0) was used to record a ^1^H,^15^N HSQC spectrum at 298 K, then FH 8–9 was added to 40 μM and the spectrum was rerecorded.

### Cross-linking and MS/MS

Samples (20 μg) of plasma-purified FH or FH plus PspCN (1:1.15 FH/PspCN molar ratio) were cross-linked with bis(sulfosuccinimidyl) suberate (BS3) using 1:0.5 or 1:4 mass ratios of protein to BS3, and an hFH concentration of 0.56 μg/μl in the reaction mixtures. Products of cross-linking were resolved by SDS-PAGE on a NuPAGE 4–12% Bis-Tris gel (Invitrogen), with MOPS running buffer, then stained with InstantBlue (Expedeon). Five excised bands corresponding to cross-linked products (see Fig. 5A) were subjected to in-gel tryptic digestion as described (42). For each sample, cross-linked peptides were fractionated using an SCX-StageTip (43); non–cross-linked peptides generally elute at 60 mM ammonium acetate whereas cross-linked peptides are enriched in the 500 mM ammonium acetate fractions. High-salt fractions were analyzed by liquid chromatography–MS/MS using a Q Exactive instrument (Thermo Fisher Scientific). Peptides were separated on an analytical column containing ReproSil-Pur C18-AQ 3 μm (Dr. Maisch, Ammerbuch-Entringen, Germany) into a PicoTip emitter (New Objective, Woburn, MA). For elution, a linear gradient was applied from 1.6 to 32% (v/v) acetonitrile in water during 109 min, followed by a rapid increase to 76% (v/v) acetonitrile. Mass spectrometric acquisitions were conducted in a data-dependent setup: following each MS scan, the 10 most intense ions were isolated and fragmented by higher energy C-trap dissociation. Both MS and MS/MS spectra were recorded in the Orbitrap analyzer with 70,000 resolution for MS scans and 35.000 for MS/MS scans. “Dynamic exclusion” was set to 60 s; “repeat count” was set to 1.

Fifty cross-linked peptides were identified using in-house software (Supplemental Table I). Label-free quantitation was performed at linkage level as follows. For each unique linkage, mass spectrometric chromatographic signal intensities of all corresponding cross-linked peptides were retrieved using Pinpoint software (Thermo Fisher Scientific) and summed. To ensure unbiased comparisons across samples, signal intensities of individual linkage pairs were normalized using the summed signal intensity of 23 cross-linked (and 29 non–cross-linked) FH peptides observed in all five samples. The intensity variation of a cross-link across the five samples was thereby reflected in its relative intensity within each sample, calculated as the logarithm to base 5 of the quotient of the observed intensity and the average intensity, of this cross-link, in five samples. Relative abundances of each cross-linked peptide among the three samples (s1, s2, and s4, as explained in *Results*) that did not contain dimeric FH were calculated. Then, for description and presentation purposes, cross-links were categorized in the following way: enriched in s1 (>65% in s1); enriched in s1 and s2 (>40% in each of s1 and s2); enriched in s2 and s4 (>40% in each of s2 and s4); enriched in s2 (>65% in s2); enriched in s4 (>65% in s4); and all the rest (present approximately equally in all three of s1, s2, and s4) (see Supplemental Table I).

The cross-linking and MS (CLMS) experiment was repeated and produced essentially the same results (not shown).

## Results

### The N-terminal domain of D39 PspCN binds effectively irreversibly to FH

**FIGURE 2.**
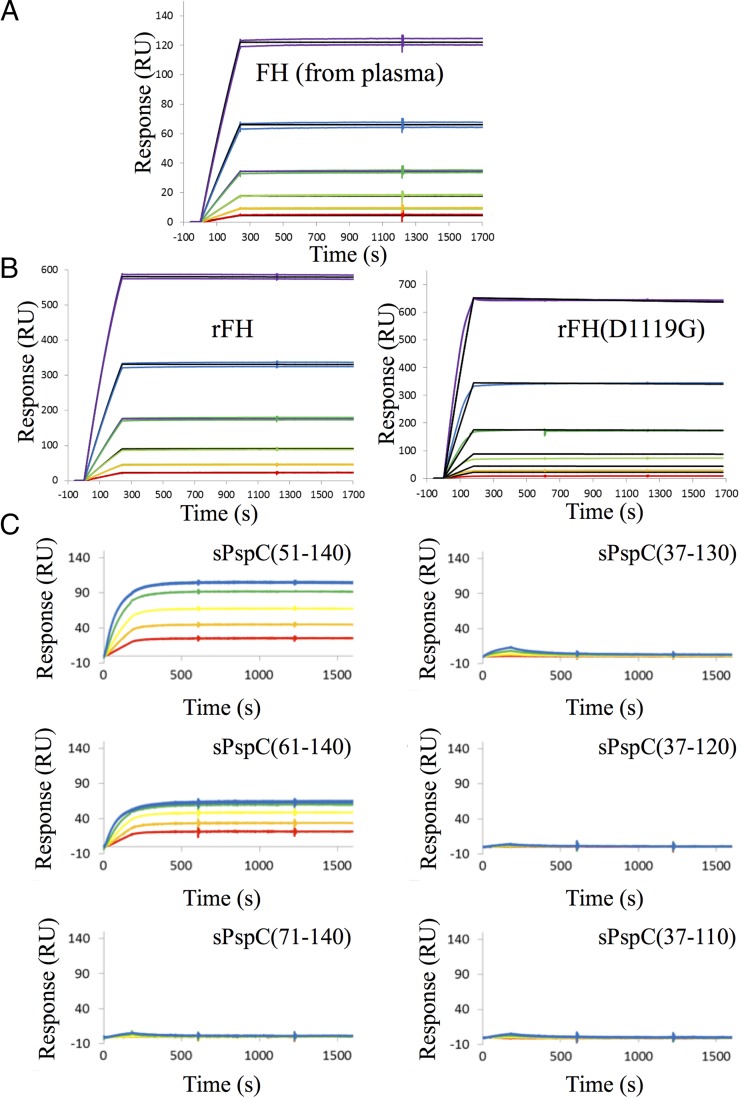
FH binds irreversibly to (D39)PspCN. (**A**) A 2-fold dilution series, from 8 to 0.25 nM, of plasma-purified human FH was flowed (in duplicate) over D39 His^6^-Sumo-PspCN immobilized (to Ni-NTA) in an SPR experiment (see also Supplemental Fig. 1). (**B**) The experiments shown in (A) were repeated with recombinant wild-type FH (*left*) or rFH(D1119G) (*right*). In (A) and (B), solid black lines indicate fitted curves. (**C**) A 2-fold dilution series from 40 to 2.5 nM of FH was flowed (in duplicate) over recombinant sPspCN truncations (as indicated using residue numbers, see Fig. 1C) immobilized on Ni-NTA. Residues 61–140 represent the minimum region of D39 PspC shown in the present study to bind tightly to FH, but (D39)PspC_37–140_ (i.e., PspCN) was used for further experiments owing to its greater stability.

The FH-binding site of the *S. pneumoniae* protein, PspC (Fig. 1C), lies within its variable N-terminal region (44–46). To explore the boundaries of this binding site and measure the affinity of the interaction, we produced His^6^-SUMO–tagged proteins corresponding to N-terminal regions of PspC from the virulent *S. pneumoniae* strain, D39. We used SPR to monitor capture of FH flowed over these proteins immobilized on a Ni^2+^-bearing sensor chip (Fig. 2A). Binding of FH to D39 PspC residues 37–140 was remarkably tight with a barely detectable off-rate (<0.2% over 30 min), equating to a pM *K*_D_. Recombinant wild-type and D1119G mutant (see below) versions of FH bound with equally strong affinities (Fig. 2B). The smallest tested D39 PspC fragment that still bound tightly to FH contained residues 61–140 (Fig. 2C). Smaller fragments were, however, less soluble after cleavage from His^6^-SUMO, so we used D39 PspCN 37–140 [henceforth (D39)PspCN] for further experiments.

### (D39)PspCN binds directly to central CCPs, but full-length FH is needed for pM binding

**FIGURE 3.**
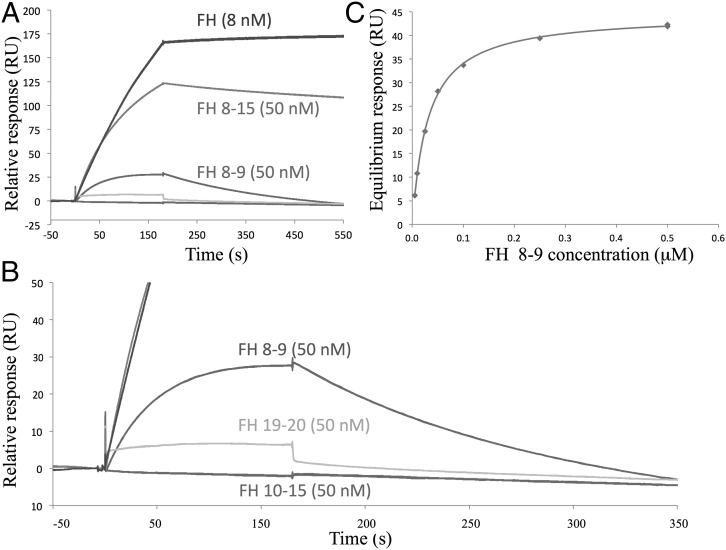
(D39)PspCN binds FH 8–9 but additional CCPs are essential for irreversible binding. D39 sPspCN was immobilized as in Fig. 2A. (**A**) Comparison of binding curves obtained for 8 nM FH as well as 50 nM FH 8–9, FH 8–15, FH 10–15 (near baseline), or FH 19–20 (just above baseline). (**B**) Expanded segment of the sensorgram in (A) highlighting small responses for FH 19–20 and FH 10–15 (*K*_D_ values could not be estimated). (**C**) An FH 8–9 concentration series was flowed over immobilized D39 sPspCN to allow estimates (equilibrium method) of *K*_D_ of 30 nM. Another series for FH 8–15 (not shown) yielded a *K*_D_ of 1 nM.

PspC from various strains of *S. pneumoniae* was reported to bind to diverse segments of FH, for example CCPs 8–11, CCPs 13–15, and CCPs 19 and 20 (44, 45, 47, 48). To further map the FH-binding site of D39 PspCN, we flowed, in an SPR experiment, our existing library of rFH fragments (16) (Fig. 1B) over immobilized (via His^6^-SUMO) (D39)PspCN (Fig. 3). On the one hand, binding of (D39)PspCN to the following fragments was undetectable or negligible: FH 1–4, FH 6–8, FH 10–11, FH 10–15, and FH 19–20. On the other hand, (D39)PspCN bound well to FH 8–9 (*K*_D_ = 30 ± 2 nM) and bound even better to FH 8–15 (*K*_D_ = 1 ± 0.1 nM). None of the fragments bound to (D39)PspCN in the effectively irreversible manner observed for intact FH.

**FIGURE 4.**
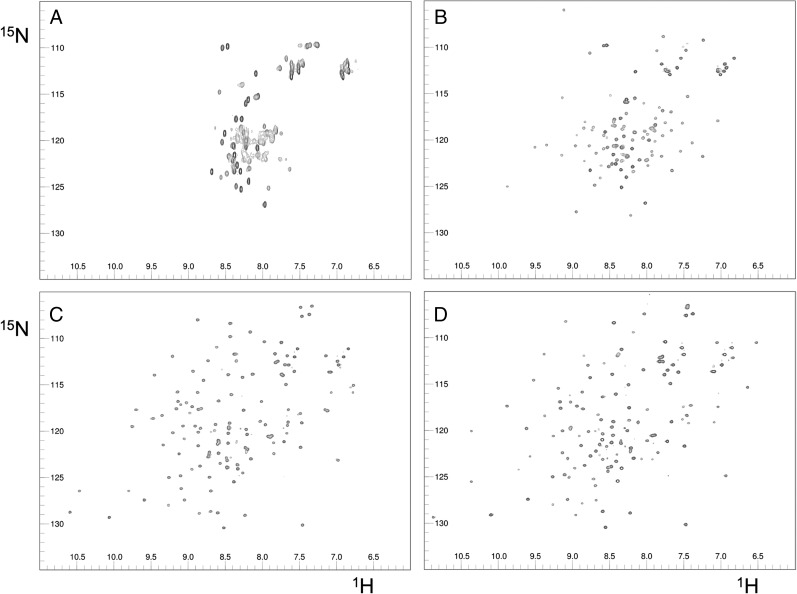
NMR-based study of FH 8-9:PspCN complex. ^1^H,^15^H(NMR) spectra for (**A**) free D39 ^15^N-PspCN, (**B**) D39 ^15^N-PspCN bound to FH 8–9, (**C**) free ^15^N-FH 8–9, (**D**) ^15^N-FH 8–9 bound to (D39)PspCN. Differences between (A) and (B) suggest D39(PspCN) adopts a more compactly folded structure upon binding FH 8–9. Many chemical shift differences between (C) and (D) suggest extensive contacts between FH 8–9 and (D39)PspCN. For higher resolution and overlaid spectra (see Supplemental Fig. 2).

To further address the participation of CCPs 8 and 9 of FH in PspCN binding, ^1^H,^15^N heteronuclear single-quantum coherence nuclear magnetic resonance (NMR) spectra of ^15^N-PspCN were recorded with and without FH 8–9 (Fig. 4A, 4B, Supplemental Fig. 2). The spectrum of (D39)PspCN suggests a molten globule as was supported by its binding of anilino-1-naphthalenesulfonic acid (data not shown). Following complex formation with FH 8–9, bound PspCN yielded a spectrum containing well dispersed and resolved cross peaks, reflecting transition to a folded conformation upon complex formation. In other experiments, spectra of ^15^N-FH 8–9 were recorded with or without unlabeled PspCN (Fig. 4C, 4D). Judging from the uniform appearance of the well-dispersed cross peaks, both modules appeared to be compactly folded with any mobility restricted to loops and turns. Fig. 4D reveals numerous chemical-shift perturbations, reflecting plentiful contacts with PspCN in CCPs 8 and 9, as may also be judged from the overlaid spectra in Supplemental Fig. 2.

**FIGURE 5.**
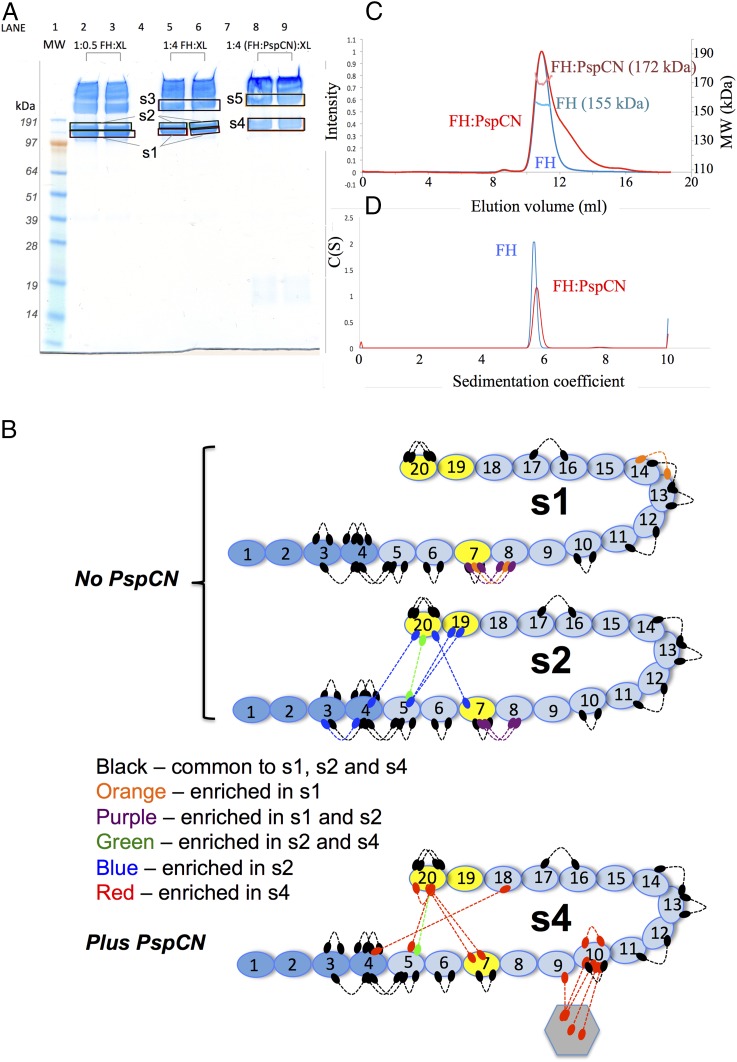
Architecture and oligomeric status of FH:PspCN complexes. (**A**) Cross-linked products were resolved by SDS-PAGE. *Lane 1*, molecular mass markers; *lanes 2* and *3*, cross-linker (BS3) mixed with FH (0.5:1 BS3/protein molar ratio); *lanes 5* and *6*, as *lanes* *2* and *3* but 4:1 BS3/protein molar ratio; *lanes 8* and *9*, BS3 mixed with FH:(D39)PspCN complex (4:1 BS3/protein complex molar ratio). Coomassie-stained bands (labeled s1–s5) were cut out for MS/MS analysis (Supplemental Table I): s1 and s2 correspond to distinct monomeric species with different migration rates, s3 corresponds to a cross-linked dimer, and s4 and s5 correspond to a cross-linked FH monomer and dimer, respectively, in complex with (D39)PspCN. (**B**) In cartoons of FH, numbered ovals indicate CCPs and dotted connectors represent cross-linked peptide pairs detected by MS/MS in analysis of SDS-PAGE bands corresponding to monomeric FH (s1 and s2) or the 1:1 FH:PspCN complex s4. Connectors are black when they represent cross-links that are observed equally in all three bands (s1, s2, and s4) or colored when they are selectively enriched in one or two bands (see key, *Materials and Methods* and Supplemental Table I). (**C**) Size-exclusion chromatograms and multiple-angle light scattering–derived molecular masses for FH and FH:(D39)PspCN complex. The 155-kDa value for FH matches the calculated monomeric molecular mass. Material in the main FH:PspCN peak had a mean estimated molecular mass of 172 kDa, close to that expected for 1:1 (167 kDa). The FH:PspCN peak has a trailing shoulder attributable to nonspecific association with resin. A minor peak (molecular mass of 189 kDa) could represent some 1:2 or 1:3 FH:PspCN complexes, but there was no evidence of complexes containing two molecules of FH (≥310 kDa). (**D**) Analytical ultracentrifugation of free FH showed a single species; FH:(D39)PspCN also behaved in analytical ultracentrifugation as a single species with a higher Svedberg value than free FH.

CLMS was used to provide additional and orthogonal evidence for which FH regions contact (D39)PspCN. The reagent BS3, which cross-links pairs of primary amines within 11.4 Å of each other, was added to mixtures of FH and PspCN. Products resolved by SDS-PAGE (Fig. 5A) were excised from the gel and trypsinized, and then MS/MS was used to identify cross-linked peptides (Supplemental Table I). Within the product of cross-linking corresponding to a 1:1 PspCN:FH complex (band s4, Fig. 5A), just five cross-links were identified between PspCN and FH, one involving CCP 9 and four involving CCP 10 (Fig. 5B).

All these data suggest that CCP 9 of FH is essential for binding (D39)PspCN. Immediately adjacent CCPs likely also contact (D39)PspCN but, in the absence of CCP 9, for example, in the contexts of FH 6–8 and FH 10–15, their affinity for (D39)PspCN was not measurable by SPR. It is also possible that CCP 10 does not contact PspCN directly but contributes to affinity by stabilizing adjacent loops in CCP 9. Direct engagement with at most three ∼6.5-kDa FH modules (CCPs 8–10) is consistent with the predicted size and hence available surface area of compactly folded 12-kDa (D39)PspCN. The extraordinarily tightness of the PspCN complex with full-length FH is noteworthy. It arises from an extremely slow off-rate, attained by full-length FH but not recapitulated by FH fragments. (D39)PspCN is likely trapped within the complex by a very stable PspCN-induced global rearrangement of CCPs. To explore further, we recorded intramolecular CLMS data for FH before and after (D39)PspCN addition.

### Analysis of FH architecture by CLMS

SDS-PAGE of FH (with no cross-linker) yielded a band commensurate with its 155-kDa molecular mass (data not shown). Inclusion of the bifunctional cross-linker BS3 yielded bands s1 and s2 (in Fig. 5A) that are consistent with a pair of differently migrating populations of conformers of cross-linked monomeric FH, as well as bands corresponding to cross-linked FH dimer (s3) and higher oligomers. Band s2 contained more head-to-tail cross-links (Fig. 5B, Supplemental Table I) than did band s1. The detection of a cross-link between side-chains in the same molecule is evidence that the two residues are proximal for at least some of the time or in at least a portion of conformers, although this cannot be quantified. The absence of a cross-link cannot, however, be used as evidence for lack of proximity because there are numerous reasons why a cross-link between two bridgeable amino groups (<11.4 Å apart in this case) might not form or might not be identified. Nonetheless, the simplest explanation for the difference between the patterns of cross-links in s1 and s2 is the presence of more bent-back FH conformations in s2 than in s1, reflecting the coexistence of both extended conformations and more compact ones in solution. The identification of multiple cross-links from CCP 20 to CCP 4, CCP5, and CCP 7 in s2 implies flexibility in the C terminus.

After addition of D39 PspCN to FH and incubation with the cross-linker, a single monomer-candidate band (s4) replaced s1 and s2, and a dimer-candidate band (s5) was enhanced (Fig. 5A). Both s4 and s5 contained PspCN cross-linked to FH CCPs 9 and 10 (as noted above). Comparison of the monomeric FH-containing bands s1, s2, and s4 (Supplemental Table I) revealed 15 differences between cross-links in the presence of PspCN (s4) compared with the absence of PspCN (s1 and s2), that is, cross-links that are selectively enriched in either or both of s1 and s2, or enriched in s4. Of these 15 cross-linked peptides, 13 involve CCPs 3–8 or CCPs 18–20 and 11 out of these 13 involve either or both of CCP 7 and CCP 20; 3 of the 15 connect CCPs 7 and 8 (Fig. 5B, Supplemental Table I). Note that the s2 (no PspCN) cross-link between CCP 7 and CCP 20 involves different residues to the two s4 (plus PspCN) cross-links between CCP 7 and CCP 20. Although cross-linking data should be interpreted cautiously, these differences strongly suggest that FH undergoes a change in conformational preferences upon binding PspCN. Moreover, the identity of the lost or gained cross-links implies that the allosteric effect of PspCN involves differences in the juxtaposition of N- (CCPs 1–7) and C-terminal (CCPs 18–20) regions of the FH molecule.

The new conformational preferences of FH induced by PspCN are likely stabilized by both intermolecular and intermodular, intramolecular contacts. These could account for the near-zero dissociation rate for this complex. PspCN-stabilized FH conformers may have nascent self-association sites, accounting for the increased intensity of the dimer band s5. Nonetheless, analysis by size-exclusion chromatography–multiple-angle light scattering (Fig. 5C) and analytical ultracentrifugation (Fig. 5D) failed to detect FH dimerization, in the absence of BS3, either before or after PspCN addition. Thus, any self-association of PspCN:FH is weak.

In short, although NMR shows that PspCN folds on binding FH, differences in cross-linking yields suggest that the conformational landscape of FH is modulated by PspCN. N- and C-terminal regions, corresponding to the principal C3b-binding segments of FH (see Fig. 1B), account for most of the differences in identified cross-links. We therefore explored the functional consequences for FH of being anchored to (D39)PspCN.

### New binding site for TED/C3d exposed by (D39)PspCN binding

**FIGURE 6.**
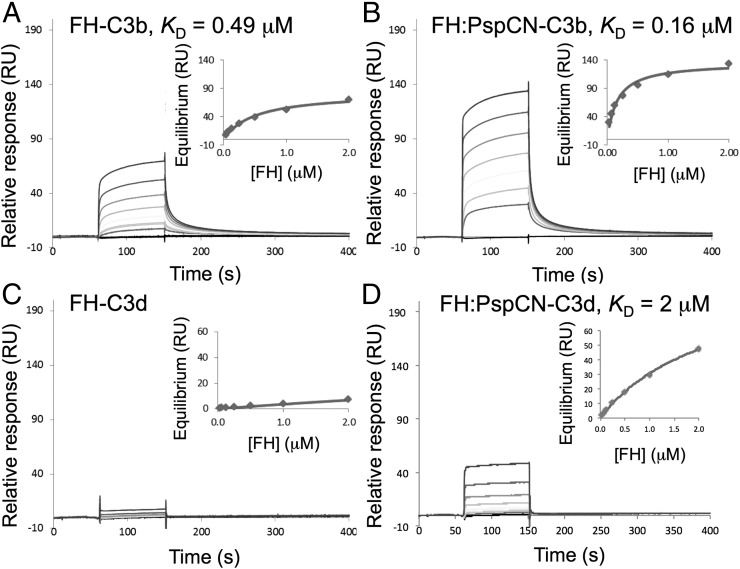
Binding properties of wild-type (plasma-derived) FH with and without D39 PspCN. (**A** and **B**) Demonstration by SPR that FH (A) has lower affinity than does the FH:(D39)PspCN complex (B) for immobilized C3b (amine coupled on a C1 chip). (**C** and **D**) The lower affinity of FH for C3b may arise from FH (C) having a very substantially lower affinity than the FH:(D39)PspCN complex (D) for immobilized C3d amine coupled to a different channel of the same C1 chip as used in (A) and (B).

Human C3b and C3d (corresponding to the TED of C3b) were amine coupled to an SPR chip. Having checked that (D39)PspCN did not bind to immobilized C3b or C3d (data not shown), the affinities of FH and the (D39)PspCN:FH complex for these C3 fragments were compared (Fig. 6). The *K*_D_ for FH:C3b, 490 nM (see Table I), matched literature values. Crucially, (D3)PspCN:FH bound tighter to C3b (*K*_D_ = 160 nM) than did FH alone (Fig. 6A, 6B). This enhancement proved to be reproducible in further measurements; in a total of 10 entirely independent measurements, the ratio of *K*_D_ values for FH versus FH-PspCN was 2.04 ± 0.56.

**Table I.** Dissociation constants for FH binding to immobilized C3b and C3d measured by SPR

Analyte	C3b				C3d			
	*K*_D_ (μM)	SE (*K*_D_)	χ^2^ (RU^2^)	*R*_max_ (RU)	*K*_D_ (μM)	SE (*K*_D_)	χ^2^ (RU^2^)	*R*_max_ (RU)
FH	0.49	0.024	0.14	76	ND	—	—	—
FH + PspCN	0.16	0.059	0.5	134	2.3	0.16	0.4	102
FH + Tigr4_37–179_	0.28	0.030	1.1	60	1.2	0.10	0.1	70
rFH	0.60	0.053	3.8	147	ND	—	—	—
rFH + PspCN	0.28	0.060	16.0	113	2.2	0.37	0.8	100
FH(D1119G)	0.54	0.093	9.4	63	ND	—	—	—
FH(D1119G) + PspCN	0.32	0.042	12.6	79	ND	—	—	—

*R*_max_, maximum response.

Alone, FH bound very poorly to C3d immobilized on the SPR chip (Fig. 6C, Table I). Although similar data may be found in the literature (25, 26, 49), this observation is remarkable given that the two-module C-terminal FH fragment, FH 19–20, binds to C3d with *K*_D_ of ∼2 μM (16, 49). Strikingly, the D39 PspCN:FH complex bound many times better to C3d than did FH alone (Fig. 6C, 6D, Table I). Indeed, the *K*_D_ of 2 μM for this interaction is reminiscent of that for the FH 19–20:C3d interaction.

In sum, the C3d-binding site (that is presumably also a C3b/TED-binding site) within the C-terminal CCPs of FH is largely inaccessible within full-length FH operating on a SPR chip. Our data show that binding of (D39)PspCN to FH exposes this C-terminal C3d/TED-binding site by remodeling FH. This allows the PspCN:FH complex (unlike FH alone) to bind well to C3d, and to bind C3b more tightly (presumably using two sites) than FH alone (using one site). We speculated that (D39)PspCN induces an “enhanced” form of FH similar to that induced by molecular markers on self-surfaces requiring protection from complement activation.

### PspCN enhances the potential of FH to regulate complement

**FIGURE 7.**
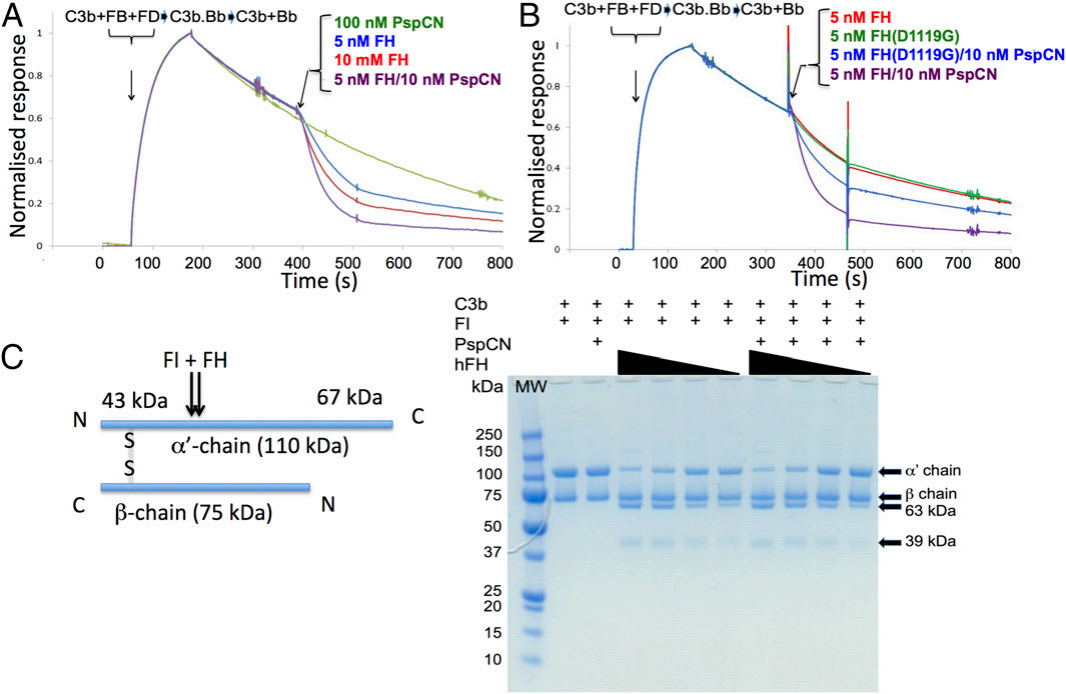
Functional properties of wild-type and mutant FH with and without D39 PspCN. (**A**) Formation of C3bBb from C3b, FB, and FD on the chip surface (see Fig. 1A) and subsequent loss of Bb, monitored by SPR. Curves are color coded (see key). Note that 100 nM PspCN alone has no activity; 5 nM FH:(D39)PspCN complex accelerated decay better than did 10 nM FH alone. (**B**) Incrementally increasing FH from 10 to 80 nM correlates with greater fluid-phase FI cofactor activity, judging by stronger bands representing the C3b α′-chain cleavage products at 39 and 63 kDa, and loss of intensity of the band corresponding to intact α′-chain. PspCN lacks intrinsic cofactor activity whereas binding of PspCN to FH does not enhance its activity. (**C**) In a parallel experiment to that of (A), 5 nM FH(D1119G) and 5 nM wild-type FH have similar DAAs but (D39)PspCN enhances the activity of wild-type FH more than it enhances the activity of FH(D1119G).

If capture by PspCN exposes a C3d/TED-binding site in FH, does it modulate the principal role of FH, that is, inhibition of the C3b-amplification loop? We measured effects of (D39)PspCN on the convertase decay-accelerating activity (DAA) of FH (Fig. 1A). To do this, we monitored C3bBb formation on the SPR chip followed by its dissociation into C3b and Bb, before and after addition of FH or (D39)PspCN:FH (Fig. 7A). Predictably, 5 and 10 nM FH dose-dependently accelerated C3bBb decay; 100 nM (D39)PspCN alone had no effect. Strikingly, 5 nM (D39)PspCN:FH was at least twice as active as 10 nM FH (alone). We next checked whether (D39)PspCN:FH has enhanced cofactor activity (Fig. 1A) by incubating C3b and FI with either FH or (D39)PspC:FH. No difference was detected (Fig. 7B). That PspCN enhances DAA and affinity for C3b of FH, but does not increase cofactor activity, is readily explicable. For example, by increasing binding of FH to iC3b, PspCN could impede product (iC3b) release (from the FH:FI:iC3b complex) and slow down turnover. Another explanation is that FI recruitment to C3b:FH (perhaps unaffected by PspCN), rather than FH binding to C3b, limits efficacy as cofactor.

### PspC from *S. pneumoniae* strain Tigr4 has similar properties to (D39)PspC

**FIGURE 8.**
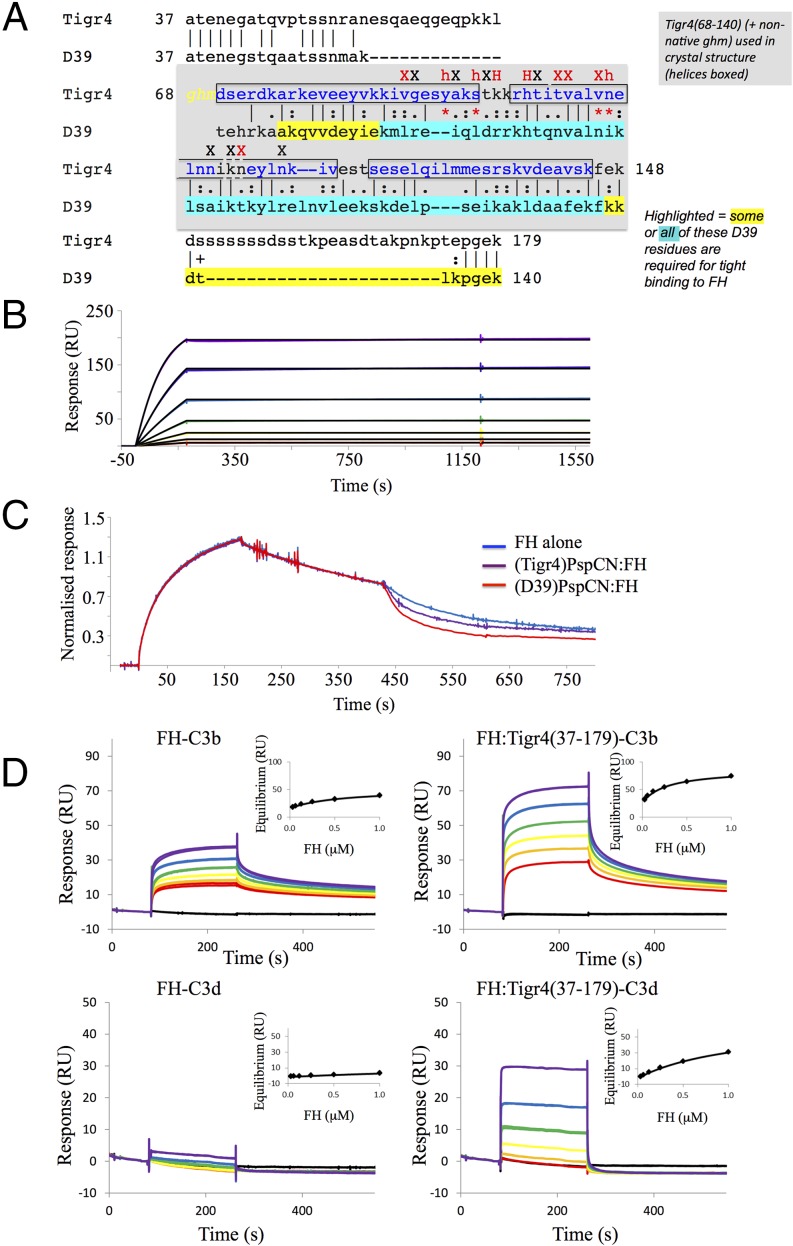
(Tigr4)PspC_37–179_ shares FH binding and activating properties with (D39)PspCN. (**A**) Pairwise (BLAST-P) alignment of N-terminal sequences from D39 and Tigr4 PspC. The sequence of (D39)PspCN (i.e., residues 37–140) is compared with that of (Tigr)PspC_37–179_. The segment of Tigr4 PspCN (residues 68–140) used in a recent crystal structure (48) of a complex with FH CCP 9 (PDB_ID = 4k12) is shaded in gray (boxes indicate α-helices); yellow and blue highlighting of residues summarizes information from truncation mutants in Fig. 2C. Intermolecular interface residues are indicated (red signifies >60% buried) as “X” or “h” (when H-bonded) or “H” if H-bonded and in salt bridge); *, key interface residue that is not conserved (analyzed using PISA) (54). (**B**) In this SPR-based experiment, a 2-fold dilution series of FH solution from 16 to 0.25 nM was flowed over immobilized (Tigr4)PspC_37–179_, suggesting that binding is irreversible. (**C**) A complex of (Tigr4)PspC_37–179_ and FH is more effective than FH alone at accelerating decay of C3bBb, but not as effective in this respect as (D39)PspCN:FH. In these experiments, 20 nM FH was used. (**D**) (Tigr4)PspCN enhances FH binding to C3b and C3d. In measurements that were analogous to those of Fig. 6, solutions of either FH (*left*), or FH:(Tigr4)PspCN_37–179_ (*right*) (at FH 2-fold dilutions from 1000 to 31 nM in both cases) were flowed over C3b or C3d amine coupled to the SPR chip. Derived *K*_D_ values are reported in Table I.

To test whether these properties of FH capture and enhancement were shared with PspC proteins from other strains of S. *pneumoniae*, we produced the N-terminal domain (residues 37–179) of PspC from the Tigr4 strain of *S. pneumoniae*, (Tigr4)PspC_37–179_, that shares 50% sequence similarity with (D39)PspCN (Fig. 8A). FH bound equally tightly to both of these proteins (Fig. 8B). Moreover, the (Tigr)PspC_37–179_:FH complex had similar properties to those of (D39)PspCN:FH in terms of enhanced C3b and C3d binding (Fig. 8D). Finally (Tigr4)PspC_37–179_ stimulated the decay-accelerating activity of FH in the same way as did (D39)PspCN (Fig. 8C).

### (D39)PspCN reveals cryptic effects of disease-linked mutation

**FIGURE 9.**
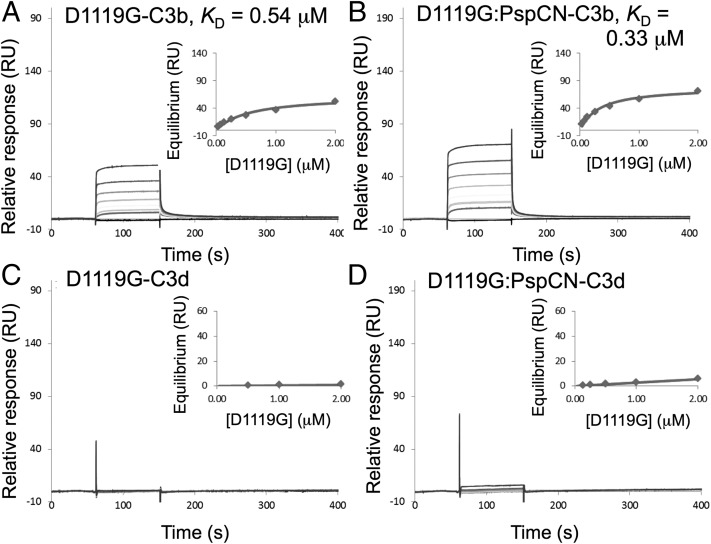
Binding properties of FH(D1119G) with and without (D39)PspCN. (**A**) According to SPR, FH(D1119G) binds to immobilized C3b with a similar *K*_D_ value to that of wild-type FH. (**B**) Importantly, there is little enhancement of C3b binding when FH(D1119G) is complexed with (D39)PspCN. Neither (**C**) FH(D1119G) alone nor (**D**) FH(D1119G):PspCN bind well to C3d. *Insets*, Plots to estimate *K*_D_s.

The results so far imply that only the N-terminal C3b-binding site of FH engages with C3b on a foreign surface such as an SPR chip. Its C-terminal C3b(TED)-binding site, we suggest, remains occluded in the absence of PspCN (or molecular markers of a self-surface). To test this, we produced rFH(D1119G). This aHUS-linked substitution has not been extensively characterized in the context of pure full-length FH. When introduced into the fragment FH 19–20, however, D1119G markedly reduced both C3d and C3b binding. Moreover, within a crystal structure of the FH 19–20:C3d complex, Asp^1119^ occupies a key position at the intermolecular interface (49). Although FH(D1119G) has a defective C-terminal C3b(TED)-binding site, we reasoned this should not impact on SPR-based measurements of binding to immobilized C3b. Indeed, we found that full-length FH(D1119G) binds to amine-coupled C3b just as tightly as does full-length wild-type FH (Fig. 9A, Table I). Thus, we conclude that the N terminus of FH dominates its interactions with C3b on a foreign or non-self surface (where its regulatory activity is not generally desirable). As expected, FH(D1119G) still bound irreversibly to PspCN (Fig. 2B) but had no measurable affinity for C3d (Fig. 9C).

We measured binding of the (D39)PspCN:FH(D1119G) complex to C3b and C3d (Fig. 9C, 9D). In contrast to wild-type FH (but in line with our model), the negligible affinity of FH(D1119G) for C3d is only enhanced slightly by (D39)PspCN (no *K*_D_ could be calculated in either case). The enhancement by PspCN of FH(D1119G) binding to C3b (*K*_D_ values of 0.33 ± 0.04 μM in the presence of PspCN versus 0.54 ± 0.09 μM in its absence) is not as clear-cut as in the case for wild-type FH. Thus, PspCN binding reveals a difference between wild-type FH and aHUS-linked FH(D1119G) that was not evident from SPR-based C3b/C3d-binding studies performed without PspCN. All these results reaffirm our hypothesis that PspCN is able to disinter the occluded C-terminal C3b(TED)/C3d–binding site of wild-type FH.

In agreement with the aforementioned retention by full-length FH(D1119G) of near–wild-type C3b-binding affinity, this mutation did not affect C3bBb DAA (in the absence of PspCN and on the foreign surface of the SPR chip) (Fig. 7C). Importantly, PspCN induced only a small improvement in the DAA of FH(D1119G), contrasting with the 5-fold improvement induced by PspCN in the case of wild-type FH. This is a strong further endorsement of our model in which the PspCN-induced improvement of DAA arises from exposure of a non-disrupted C-terminal C3b/C3d-binding site.

**FIGURE 10.**
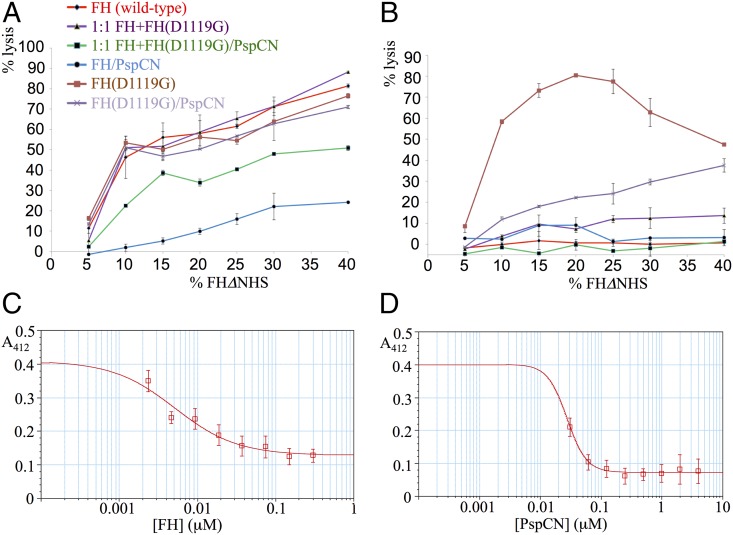
Cell-based assays. (**A**) Near-complete human complement-mediated lysis (after 30 min) of rabbit erythrocytes, measured by A_412_ of the released hemoglobin at 40% (v/v) FHΔNHS. Neither FH, nor FH(D1119G), nor 1:1 FH+FH(D1119G) protected rabbit erythrocytes significantly. (D39)PspCN:FH(wild-type), but not (D39)PspCN:FH(D1119G), affords some, although incomplete, protection. (**B**) As expected, FH prevents human complement-mediated hemolysis of (human-like) sheep erythrocytes irrespective of (D39)PspCN, whereas FH(D1119G) provides less protection than does wild-type FH. Protection of sheep erythrocytes by the 1:1 mixture of FH(wild-type) plus FH(D1119G), recapitulating heterozygosity, is enhanced by PspCN. (**C** and **D**) (D39)PspCN boosts ability of FH in serum to prevent hemolysis of PNH-like human erythrocytes (treated with AET to deprive them of decay-accelerating factor). These AET-treated cells are susceptible to acidified serum lysis in a similar way to erythrocytes from PNH patients. (C) Adding increasing concentrations of FH inhibits lysis of the PNH-like erythrocytes. (D) Adding increasing concentrations of (D39)PspCN inhibits lysis of the PNH-like erythrocytes with an IC_50_ of 28 nM.

We also investigated whether the aHUS-linked D1119G substitution perturbs the regulatory activity of FH on a surrogate host-cell surface, as opposed to the foreign surface of an SPR chip. In control assays, addition of wild-type FH to FHΔNHS did not afford much protection to rabbit erythrocyte surfaces that lack human-like sialic acids (Fig. 10A). Alternatively, and concordant with classical studies (50), sheep erythrocytes, which are rich in human-like sialic acids, were protected by FH from complement-mediated hemolysis (Fig. 10B). Crucially, FH(D1119G) protected neither rabbit nor sheep erythrocytes. Thus, despite FH (wild-type) binding to immobilized C3b, and decaying C3bBb no better than FH(D1119G) on the “foreign” surface of a SPR chip, the wild-type protein functions far better than the mutant on a surrogate host-cell surface. This is in agreement with our hypothesis that FH binding to C3b-bearing self-surfaces (but not foreign ones such as the SPR chip surface) induces exposure of the C-terminal C3b(TED)-binding site of wild-type FH that is missing in FH(D1119G).

### (D39)PspCN can rescue complement-regulation deficiency

**FIGURE 11.**
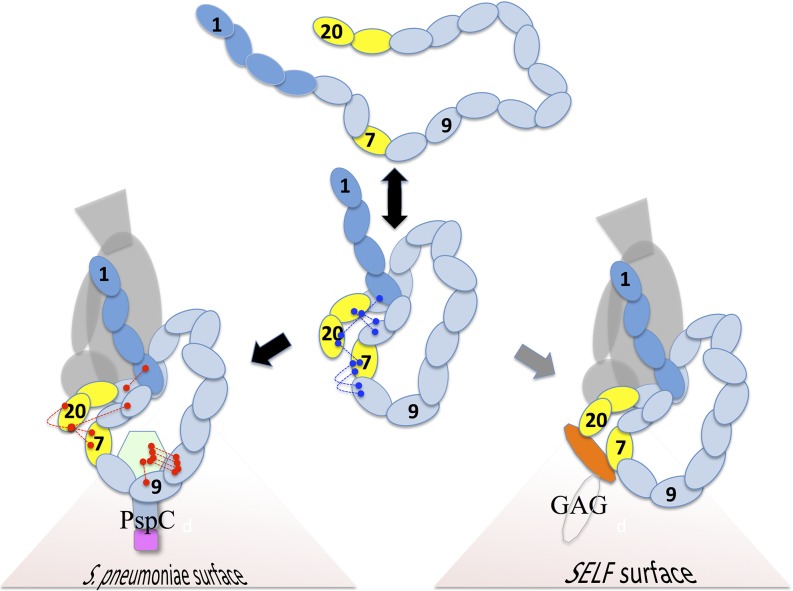
Model for functional enhancement of FH by self-surface markers and PspCN. Ovals represent CCPs; selected cross-links are shown (see Fig. 5B). Circulating FH (*center panels*) adopts extended conformations (from band s1 in Fig. 5A), not conducive to cross-links between non-neighboring CCPs. Also adopted are more compact conformations that are captured by cross-linking (from band s2 in Fig. 5A). We cannot tell which of these prevail under physiological conditions. PspCN stabilizes FH conformers that resemble the bent-back ones in s2 but that produce different cross-linking patterns. The PspCN-stabilized FH conformers (*bottom left*) bind better to C3b and C3d. Rearrangements of CCPs 7 and 20 probably contribute to functional enhancement, as this is where most differences in cross-linking occur. These two CCPs also bind to GAGs and sialic acid and to numerous other bacterial proteins. This suggests that engagement with these markers of self-surfaces may induce a functionally enhanced conformation of FH (*bottom right*) similar to the one captured by cross-linking in band s4 of Fig. 5A.

The PspCN:FH(wild-type) complex failed to fully protect rabbit erythrocytes, used herein as surrogate foreign cells (Fig. 10A). This is despite the enhanced C3b/C3d-binding and decay-accelerating activity of the complex that was detected using SPR. An additional requirement for protection of bacteria is likely to be localization of PspC on the bacterial surface (via the choline-binding domain that is absent from PspCN) (see model in Fig. 11).

We partially recreated the scenario of a heterozygous individual with the FH(D1119G) mutation by performing hemolysis assays using a 1:1 mixture of wild-type FH and FH(D1119G). In our assay, this mixture did not completely protect (human-like) sheep erythrocytes from complement-mediated lysis (see FH+FH(D1119G) in Fig. 10B). When PspCN was also included with the FH/FH(D119G) mixture, no hemolysis could be detected. Moreover, soluble (D39)PspCN boosted the ability of FH(D1119G) (in the absence of wild-type FH) to protect (human-like) sheep erythrocytes from complement-mediated lysis (Fig. 10B). This ability of PspCN to partially rescue the regulatory activity of FH(D1119G) could reflect the residual C3d binding of the mutated C terminus that might also explain the small enhancements of C3b binding and DAA induced in FH(D1119G) by PspCN.

Finally, we modeled PNH, wherein a somatic mutation results in erythrocytes that lack the GPI-linked complement regulators, decay-accelerating factor (CD55) and CD59 (that inhibits formation of the membrane-attack complex, see Fig. 1A). The absence of these regulators is linked to complement-mediated lysis of erythrocytes in these patients. FH and FH-inspired candidate therapeutics protect erythrocytes from PNH patients (25), as well as erythrocytes that have been stripped of GPI-linked proteins and rendered “PNH-like” by treatment with 2-aminoethylisothiouronium bromide (39). We demonstrated (Fig. 10C, 10D) dose-dependent protection of PNH-like erythrocytes by (D39)PspCN acting in partnership with FH.

## Discussion

Building on previous work by others (31, 44), we showed that recombinant N-terminal segments of both *S. pneumoniae* D39 PspC and Tigr4 PspC, sharing 50% sequence similarity, form exceptionally stable complexes with FH. Resolving ambiguities in past literature (44, 45, 47), we showed that (D39)PspCN binds exclusively within CCPs 8–10 of FH. This agrees with a recent report that a recombinant fragment (Tigr4)PspC_68–148_ [somewhat smaller than our recombinant fragment (Tigr4)PspC_37–179_] binds mainly to CCP 9 (48) (see below). (D39)PspCN undergoes folding upon binding, whereas free (Tigr4)PspC_68–148_ exists as a three-helical bundle. Strikingly, both the D39 and Tigr4 proteins bind full-length FH two orders of magnitude more tightly than they bind to any fragments of FH. This strongly implicates FH modules other than the directly interacting ones in stabilization of the complex with full-length protein. Attachment by central modules to PspC likely induces formation of conformers that are not readily kinetically available to free FH.

The recently reported crystal structure (PDB_ID = 4k12) of Tigr4 PspC_68–148_ bound to FH 9 (48) reveals that 4 of 10 critical interface residues in this complex are not conserved in (D39)PspCN (see Fig. 8A). Missing residues include TigR Tyr^90^, the “key inserted into a lock” of hydrophobic side chains within FH 9, “latched” by an H-bond to the FH 9 backbone. Clearly, D39 PspC and Tigr4 PspC must interact with different side chains within CCPs 8–10 of FH. Despite this, both have the same remarkable FH-anchoring and function-enhancing capabilities. This supports our theory that these bacterial proteins exploit an inherent propensity of the FH molecule to switch to its enhanced conformation.

Cross-linking suggests (D39)PspCN increases FH dimerization. No dimers could be detected by analytical ultracentrifugation or multiple-angle light scattering. Nonetheless, PspCN-facilitated conformational rearrangements could expose previously buried FH regions that then favor self-association. Soluble GAG surrogates, such as heparin, reportedly cause soluble FH to oligomerize (51). Thus, in solution, both GAG:FH and PspCN:FH complexes may self-associate, albeit weakly, via newly exposed interaction sites (see Supplemental Table I). Whether this happens on surfaces is unproven.

FH interacts well with C3b (*K*_D_ of ∼500 nM) immobilized on an SPR chip but poorly with C3d (i.e., the TED of C3b) in the same context, as was also reported previously (25, 52). However, FH 19–20 (lacking CCPs 1–18) bound well to both C3b and C3d (*K*_D_ of ∼2 μM) (49). Clearly the FH C terminus is not fully competent for binding C3d on this “foreign” (and therefore complement-activating) chip surface. This binding site remains unavailable or underutilized when FH interacts with similarly immobilized C3b, explaining our critical observation that FH(D1119G) and wild-type FH bind C3b equally well, despite partial disruption of the TED/C3d-binding site in FH(D1119G) (49).

In contrast, the binding site for TED/C3d is fully available in wild-type FH complexes with (D39)PspCN and (Tigr4)PspC_37–179_. This is the simplest explanation for the massively enhanced binding to surface-immobilized C3d of these complexes compared with intact FH. Indeed (D39)PspCN:FH and (Tigr4)PspC_37–179_:FH both bind C3d on the SPR chip just as well as does FH 19–20. Simultaneous binding to C3b by both CCPs 1–4 and CCPs 19 and 20 of PspCN-bound FH explains its 2-fold higher affinity (than FH alone) for C3b. This modest affinity difference could be critical for survival of the bacterium in the host. Together with the marked improvement in DAA, it could maintain levels of bacterial surface-tethered C3b molecules below the threshold for run-away activation via the positive-feedback amplification loop. This would explain the improved hemolysis prevention properties of soluble PspCN-bound FH (Fig. 10). The capacity of PspCN to restore hemolysis protection in FHΔNHS supplemented with a 1:1 mixture of wild-type FH and FH(D1119G) hints at therapeutic potential as does the enhancement by PspCN of FH-mediated protection of PNH-like erythrocytes that lack decay-accelerating factor and CD59.

Most (11 of 15) of the differences in BS3–cross-linked peptides found between FH before and after PspCN binding involve CCPs 7 and/or 20. Many bacterial FH-binding proteins (unlike PspC) bind to both CCPs 7 and 20, potentially bringing them together. This seems unlikely to be coincidental. It suggests other bacterial complement-evasion proteins induce a broadly similar activated FH conformation, albeit via a different mode of FH engagement. Moreover, polyanionic self-surface markers also bind both CCPs 7 and 20 (16) (Fig. 11) as do oxidation-specific epitopes used for recruiting FH to damaged cells (53). An intriguing possibility is therefore that markers on self-surfaces also stabilize this, or broadly similar, functionally enhanced conformations of FH, that is, conformations characterized by an exposed TED/C3d-binding site.

In conclusion, our evidence suggests that latent FH circulates with some of its C3b-binding capabilities effectively locked away, restricting its ability to regulate C3b amplification in fluid phase or when passively captured by bacteria. FH also exists in a functionally enhanced form, with both C3b-binding sites fully competent, allowing binding to C3d and avid binding to C3b or (presumably) C3bBb (Fig. 11). Tigr4 and D39 PspC both exploit the potential existence of this functionally enhanced conformation and probably other bacterial FH-binding proteins do as well. That bacteria are able to induce a functionally enhanced conformation of FH suggests self-surfaces do likewise. Thus, PspCN has likely revealed the molecular basis of self versus non-self discrimination by the key soluble regulator of the complement system.

## Supplementary Material

Data Supplement
