# Supplementary material for: Complement Evasion Mediated by Enhancement of Captured Factor H: Implications for Protection of Self-Surfaces from Complement
Source: J Immunol. 2015 Oct 12;195(10):4986–98. doi: 10.4049/jimmunol.1501388 (PMC4635569; doi:10.4049/jimmunol.1501388)
Supplement: Data Supplement [file JI_1501388.zip › JI_1501388_Supplemental_Material_1.pdf]

Complement evasion mediated by enhancement of captured factor H - implications for protection of self-surfaces from complement

Andrew. P. Herbert, Elisavet Makou, Zhuo A. Chen, Heather Kerr, Anna Richards, Juri Rappsilber and Paul N. Barlow

SUPPLEMENTARY INFORMATION

Contents:

Supplementary Table:
Data from cross-linking and mass spectrometry analysis of FH amd FH:PspCN complex

Supplementary Figure 1:
Interaction of FH and PspCN measured by SPR (data before and after subtraction)

Supplementary Figure 2:
Expanded and overlaid HSQC spectra for (D39)PspCN and FH 8-9

Supplementary Table. Data from cross-linking and mass spectrometry analysis of FH amd FH:PspCN complex

Cross-linked residues				Abundance (peak area)					Relative abundance[4]		
Residue I (CCP)	***Residue ii (CCP)***	n[1]	XL[2]	s1[3]	s2	s3	s4	s5	s1	s2	s4
**ENRICHED in s1**											
**FH 784 (CCP13)**	**FH 854 (CCP14)**	1	1	5.6E+07	7.7E+06	0	0	0	88%	12%	0%
**FH 424 (CCP7)**	**FH 470 (CCP8)**	2	2	6.4E+08	2.3E+08	0	0	0	73%	27%	0%
**ENRICHED in s2**											
**FH 183 (CCP3)**	**FH 211 (CCP4)**	2	2	3.4E+08	5.5E+08	0	0	0	38%	62%	0%
**FH 211 (CCP4)**	**FH 1186 (CCP20)**	2	1	1.6E+07	1.7E+08	2.6E+07	4.8E+07	1.5E+07	7%	73%	20%
**FH 308 (CCP5)**	**FH 1108 (CCP19)**	1	1	7.5E+06	7.5E+07	0	0	0	9%	91%	0%
**FH 308 (CCP5)**	**FH 1148 (CCP19)**	1	1	9.8E+06	7.2E+07	0	0	5.8E+07	12%	88%	0%
**FH 410 (CCP7)**	**FH 1202 (CCP20)**	2	1	0	1.3E+07	8.8E+06	0	3.9E+07	0%	100%	0%
**ENRICHED in s4**											
Intra-PspCN											
**PspCN 26**	**PspCN 74**	2	1	0	0	0	1.5E+07	1.1E+07	0%	0%	100%
**PspCN 35**	**PspCN 76**	9	2	0	0	0	9.6E+08	6.8E+08	0%	0%	100%
**PspCN 46**	**PspCN 100**	13	2	0	0	0	3.2E+09	6.0E+09	0%	0%	100%
**PspCN 48**	**PspCN 100**	3	2	0	0	0	2.6E+09	5.0E+09	0%	0%	100%
**PspCN 56**	**PspCN 100**	9	2	0	0	0	2.0E+09	2.7E+09	0%	0%	100%
**PspCN 56**	**PspCN 86**	37	2	0	0	0	1.5E+08	8.5E+08	0%	0%	100%
**PspCN 63**	**PspCN 74**	8	1	0	0	0	2.4E+09	3.0E+09	0%	0%	100%
PspCN-FH											
**FH 520 (CCP9)**	**PspCN 26**	5	1	0	0	0	1.1E+09	7.0E+08	0%	0%	100%
**FH 584 (CCP10)**	**PspCN 100**	5	1	0	0	0	2.2E+08	5.8E+07	0%	0%	100%
**FH 588 (CCP10)**	**PspCN 100**	7	4	0	0	0	2.2E+09	2.4E+09	0%	0%	100%
**FH 588 (CCP10)**	**PspCN 46**	2	2	0	0	0	6.2E+07	1.3E+08	0%	0%	100%
**FH 588 (CCP10)**	**PspCN 48**	1	1	0	0	0	1.3E+07	4.8E+07	0%	0%	100%
Intra FH											
**FH 236 (CCP4)**	**FH 1066 (CCP18)**	5	1	0	0	1.8E+08	1.1E+08	1.7E+08	0%	0%	100%
**FH 308 (CCP5)**	**FH 1202 (CCP20)**	14	1	1.2E+08	6.1E+08	3.5E+09	1.6E+09	3.0E+09	5%	26%	69%
**FH 388 (CCP7)**	**FH 1202 (CCP20)**	1	1	0	2.2E+07	2.5E+07	6.5E+07	1.6E+08	0%	25%	75%
**FH 405 (CCP7)**	**FH 1202 (CCP20)**	15	1	3.2E+07	2.9E+08	8.0E+08	9.5E+08	6.4E+08	3%	23%	75%
**FH 573 (CCP10)**	**FH 583 (CCP10)**	9	1	6.3E+07	5.5E+07	1.7E+09	8.8E+08	7.1E+08	6%	5%	88%
**FH 1170 (CCP20)**	**FH 1202 (CCP20)**	1	1	0	0	0	1.2E+08	1.5E+08	0%	0%	100%

**ENRICHED in s1 and s2**											
**FH 424 (CCP7)**	**FH 446 (CCP8)**	27	2	1.4E+10	1.3E+10	0	3.3E+09	0	47%	42%	11%
**FH 424 (CCP7)**	**FH 496 (CCP8)**	13	2	2.4E+09	2.2E+09	2.3E+08	1.7E+08	1.7E+08	50%	46%	4%
**ENRICHED in s2 and s4**											
**FH 308 (CCP5)**	**FH 1186 (CCP20)**	7	2	5.5E+07	2.5E+08	5.2E+08	2.3E+08	5.2E+08	10%	47%	43%
**NO SIGNIFICANT CHANGE**											
**FH 183 (CCP3)**	**FH 204 (CCP3)**	29	2	7.9E+08	1.1E+09	6.6E+08	1.9E+09	2.7E+09	21%	30%	50%
**FH 204 (CCP3)**	**FH 228 (CCP4)**	14	1	2.1E+08	1.4E+08	4.3E+08	4.6E+08	4.1E+08	26%	17%	57%
**FH 211 (CCP4)**	**FH 228 (CCP4)**	54	4	6.4E+09	1.0E+10	2.1E+10	2.6E+10	1.1E+10	15%	24%	61%
**FH 224 (CCP4)**	**FH 236 (CCP4)**	15	2	5.1E+08	2.9E+08	1.1E+09	8.7E+08	1.1E+09	31%	18%	52%
**FH 236 (CCP4)**	**FH 265 (CCP5)**	51	2	1.1E+10	9.0E+09	6.0E+09	1.3E+10	2.3E+10	33%	27%	40%
**FH 236 (CCP4)**	**FH 308 (CCP5)**	48	3	3.1E+09	2.2E+09	2.9E+09	3.6E+09	4.6E+09	35%	25%	40%
**FH 265 (CCP5)**	**FH 308 (CCP5)**	23	3	4.2E+09	3.9E+09	2.9E+09	6.7E+09	8.6E+09	28%	27%	45%
**FH 327 (CCP6)**	**FH 336 (CCP6)**	2	1	1.2E+09	1.7E+09	3.8E+09	3.8E+09	3.6E+09	18%	25%	57%
**FH 388 (CCP7)**	**FH 405 (CCP7)**	14	1	4.2E+08	7.0E+08	7.3E+08	1.6E+09	1.6E+09	15%	25%	59%
**FH 573 (CCP10)**	**FH 584 (CCP10)**	69	3	2.7E+10	2.3E+10	9.1E+10	8.0E+10	7.9E+10	21%	18%	62%
**FH 642 (CCP11)**	**FH 739 (CCP12)**	30	7	1.0E+10	1.1E+10	2.7E+09	6.4E+09	9.9E+09	38%	39%	23%
**FH 754 (CCP13)**	**FH 769 (CCP13)**	118	6	8.9E+10	7.4E+10	1.9E+10	3.1E+10	2.3E+10	46%	38%	16%
**FH 979 (CCP16)**	**FH 987 (CCP17)**	2	1	2.3E+08	1.0E+08	1.3E+08	2.3E+08	2.3E+08	41%	18%	41%
**FH 1186 (CCP20)**	**FH 1202 (CCP20)**	27	2	1.6E+10	1.7E+10	6.2E+09	1.9E+10	1.0E+10	31%	33%	36%
**FH 1202 (CCP20)**	**FH 1230 (CCP20)**	14	3	1.6E+09	2.2E+09	1.0E+09	2.8E+09	1.2E+09	25%	33%	42%
**UNIQUE to BANDS CONTAINING FH DIMERS**											
**FH 308 (CCP5)**	**FH 308 (CCP5)**	7	1	0	0	9.3E+06	0	4.0E+07	0%	0%	0%
**FH 308 (CCP5)**	**FH 323 (CCP5)**	3	1	0	0	6.1E+08	0	2.6E+08	0%	0%	0%
**FH 308 (CCP5)**	**FH 388 (CCP7)**	3	1	0	0	2.0E+08	0	7.7E+08	0%	0%	0%
**FH 323 (CCP5)**	**FH 388 (CCP7)**	1	1	0	0	8.2E+06	0	4.1E+07	0%	0%	0%
**FH 308 (CCP5)**	**FH 405 (CCP7)**	7	1	0	0	2.5E+09	0	1.9E+09	0%	0%	0%
**FH 1066 (CCP18)**	**FH 1202 (CCP20)**	3	1	0	0	6.4E+07	0	7.1E+07	0%	0%	0%

[1] *n* = number of times identified in MS 2
[2] XL = number of quantified cross-linked peptides
[3] s1 to s5 = bands cut out of gel – see Figure 5 for key
[4] Relative abundance amongst s1, s2 and s4 only (these correspond to bands in which there is no FH dimer, PspCN is present in s4)

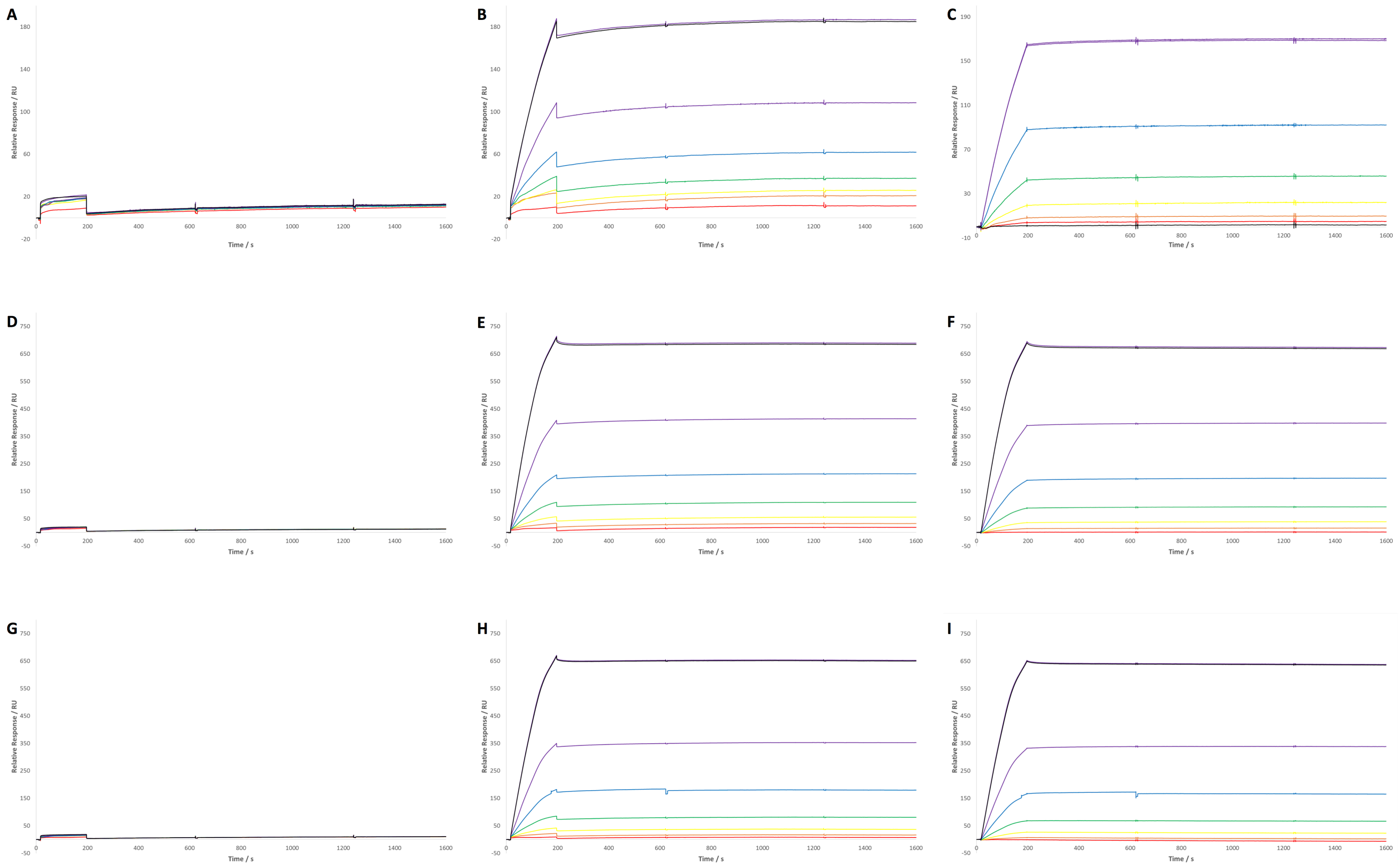

SUPPLEMENTARY FIGURE 1

Interaction of FH and PspCN measured by SPR (data before and after subtraction)

(A) Plasma-purified FH flowed over the blank (no PspCN) Ni^{2+}/nitrilotriacetic acid (NTA) chip surface in flow-cell 1. A short-lived interaction between FH and the blank surface is evident, probably mediated by the metal ions. (B) Plasma-purified FH flowed over the PspCN-loaded NTA chip surface in flow-cell 2. (C) The result of subtracting the data collected in flow-cell 1 from date recorded in flow-cell 2. (D), (E) and (F) Equivalent data to that displayed in the first three panels (*i.e.* flow-cell 1, flow-cell 2, and flow-cell 2-flow-cell 1, respectively) but in this case, for recombinant FH. (G), (H) and (I) Equivalent data to the three previous panels (*i.e.* flow-cell 1, flow-cell 2, and flow-cell 2 minus flow-cell 1, respectively) but in this case for recombinant (D1119G)FH.

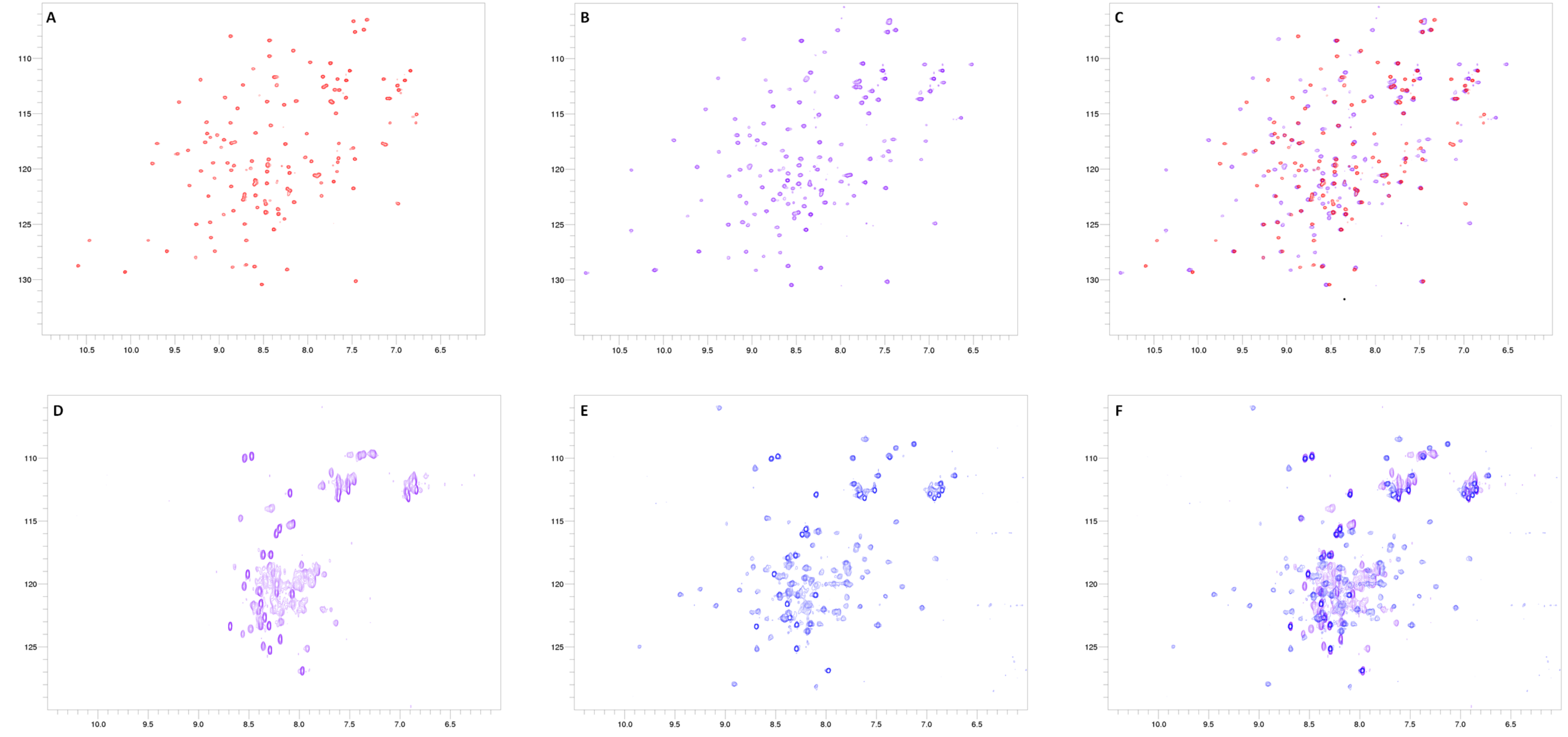

SUPPLEMENTARY FIGURE 2

Expanded and overlaid HSQC spectra for (D39)PspCN and FH 8-9

As described in the text, $^{1}H,^{15}N$ HSQC spectra were collected and are presented as follows: (A) ^{15}N FH 8-9; (B): ^{15}N FH 8-9 + (non-labeled) PspCN; (C) Overlay of (A) and (B); (D) ^{15}N PspCN; E: ^{15}N PspCN + (non-labeled) FH 8-9 (F) Overlay of (D) and (E).
